# How to use thionamide anti-thyroid drug in the young– what’s new?

**DOI:** 10.1186/s13044-021-00109-x

**Published:** 2021-07-21

**Authors:** Tim Cheetham

**Affiliations:** 1 Faculty of Medical Sciences, Translational and Clinical Research Institute, Newcastle University, Newcastle upon Tyne, NE2 4HH UK; 2 Department of Paediatric Endocrinology, Royal Victoria Infirmary, Great North Children’s Hospital, Newcastle upon Tyne, NE1 4LP UK

**Keywords:** Graves’ disease, Childhood, Anti-thyroid drug, Block and replace, Dose titration

## Abstract

The excess thyroid hormone secretion that characterises Graves’ disease (GD) is generated when stimulatory antibodies bind to the thyroid stimulating hormone receptor on the follicular cell of the thyroid gland.

This underlying mechanism cannot easily be abolished and the mainstay of Graves’ disease (GD) management in the young remains thionamide anti-thyroid drug (ATD). Unfortunately, GD will usually recur after a 2 or 3 year course of ATD, even when the stimulatory antibody titres have fallen. The diagnosis of GD therefore usually signals the start of a lengthy period of out-patient assessments and associated venepuncture. Careful, more protracted administration of ATD may increase the likelihood of longer-term remission and reduce the likelihood of the patient developing ATD side-effects. An understanding of how best to use ATD and an awareness of the less well-known consequences of GD and its’ treatment - such as excessive weight-gain and long-term hypothyroidism – are also of fundamental importance.

Recent clinical studies have shed light on how best to manage the young patient with GD and the associated new information will help to answer some of the questions posed by the young person and their family at diagnosis. This new knowledge is the focus of this article about ATD therapy in the young.

## Introduction

This article will discuss the use of antithyroid drug (ATD) treatment in the young person with Graves’ disease (GD). This is because there have been a number of pertinent publications in recent years that can help paediatricians and paediatric endocrinologists to use ATD effectively. This article will highlight many of these recent, relevant articles.

The focus of this article will be GD although it is important to note that there are other, rarer causes of thyroid hormone excess in children – such as McCune Albright syndrome or hyperthyroidism due to activating TSH-receptor mutations - that have a different natural history but that may also benefit from ATD therapy. The management of transient neonatal thyrotoxicosis due to transplacental passage of thyroid stimulating hormone receptor antibodies (TRAb) from a mother with GD will also frequently involve the use of ATD but will not be discussed in this article either.

### What is Graves’ disease?

GD is an autoimmune disorder characterised by increased levels of pathogenic thyroid stimulating hormone receptor antibodies (TRAb) to the thyroid stimulating hormone receptor (TSHR) on the thyroid follicular cell (also called thyroid epithelial cell), resulting in thyroid gland hyperfunction. Many patients have thyroid peroxidase (TPO) antibodies as well but these are not the main driver behind the excess thyroid hormone production in this context. GD is uncommon in children, affecting 1 to 3 per 100,000 individuals per year under the age of 15 [1]. The female predominance that characterises most autoimmune conditions is observed in GD although this is less pronounced in the younger child than in the adolescent. A number of genetic loci (for example thyroid stimulating hormone receptor, TSHR and Cytotoxic T-lymphocyte-associated protein 4, CTLA4) are implicated in GD susceptibility and are typically immunoregulatory in nature (for example CTLA-4) or involve thyroid autoantigen (for example TSHR) [2, 3, 4]. Environmental factors (for example smoking and iodine status) are also associated with GD susceptibility and development [5]. There is evidence to suggest that GD is becoming more common in the young although the reasons for this are not clear [6–8].

### What is different about the young patient presenting with GD?

Younger GD patients tend to have more severe hyperthyroidism at presentation when compared to adults [9] and this may be partly related to the nature of the underlying immune dysfunction. Recent data have shown that the human leukocyte antigen (HLA) complex P5 (HCP5) polymorphism is independently associated with GD susceptibility and age of onset [10].

Thyroid dysfunction may not immediately spring to mind as a potential diagnosis in the young child with GD and referral to a range of different sub-specialists is not uncommon pre-diagnosis. Patients may have prominent gastrointestinal upset, palpitations, behavioural change including anxiety and some may have abnormal development and struggle at school. Patients with GD do not always have a history of weight loss at diagnosis either and many do not have a noticeable goitre. A link between thyroid status and educational / behavioural difficulties at school or at home may be explained in other ways before thyroid function is checked and a diagnosis of GD reached [11].

### Diagnosing GD

The key biochemical and immunological picture in the patient with GD is a suppressed thyroid stimulating hormone (TSH) level (below the assay threshold) in the presence of raised thyroid hormone concentrations (FT4 and FT3). An elevated FT3 level is a more sensitive marker of GD than an elevated FT4. A raised thyroid receptor antibody (TRAb) titre is the key immunological hallmark of GD and thyroid peroxidase (TPO) antibodies will frequently be elevated as well. If the clinical picture is suggestive of GD, but thyroid antibodies are absent, then antibodies should be repeated after a few weeks. In the interim thyroid ultrasonography and scintigraphy may be useful; thyroid ultrasonography with Doppler blood flow assessment will suggest gland hyper-function in GD and scintigraphy with an agent such as technetium will show generalized, enhanced uptake. These imaging modalities will also help to exclude non-autoimmune causes of hyperfunction such as a ‘hot’ autonomous nodule.

### ATD and the young patient

Once a diagnosis of GD has been made then the key therapeutic intervention in the great majority of patients is thionamide ATD. The thionamides’ Carbimazole (CBZ) or Methimazole (MMI) have been the key treatment for the young person with newly diagnosed GD for over 60 years. Following absorption CBZ is hydrolysed to form the active component MMI; 1.0 mg of CBZ is approximately equivalent to 0.6 mg of MMZ. CBZ and MMI need only be given once daily. The thionamide propylthiouracil (PTU) – which has to be administered several times each day - is no longer routinely used in the young person because of the risk of acute hepatic failure [12] and there are few occasions when PTU use can be justified.

### ATD mechanism of action

Thionamide’s primary mode of action is to inhibit the oxidation of iodide and the organification of iodine by inhibiting the TPO enzyme which will then prevent iodination of tyrosine residues onto the thyroglobulin molecule. PTU also inhibits type 1 iodothyronine deiodinases and hence the peripheral conversion of T4 to T3. The major reason why TRAb levels fall on ATD is most likely linked to altered antigen presentation although a direct immunomodulatory action has also been proposed and there is in vitro evidence of thionamide inhibiting thyroid autoantibody production in cultured lymphocytes and in vivo evidence of increased suppressor T cells and decreased intrathyroidal activated T cells [13]. The increased basal metabolic rate in GD is also associated with the production of reactive oxygen species (ROS) and oxidative stress [14]. This may be particularly important in Graves’ orbitopathy (GO) where ROS may have a central pathogenic role [15]. ATD may possess antioxidant activity by reducing the production of ROS from white blood cells [16].

### Initial treatment

Newly presenting patients can be managed according to their symptoms, signs and biochemistry. Once the diagnosis of GD has been confirmed CBZ should be started in a dose of 0.5 to 0.75 mg/kg (MMI 0.3 to 0.5 mg/kg/day) per day. Young patients with a milder clinical and biochemical picture can be a commenced on a lower CBZ dose of 0.25 to 0.5 mg/kg per day (MMI 0.2 to 0.3 mg/kg/day). A beta blocker such as propranolol or atenolol can be administered in the more profoundly symptomatic patient provided there are no contra indications such as asthma. The beta blocker can be stopped when thyroid hormone concentrations have fallen to within or close to the reference range. It is important to remember that TSH concentrations can be suppressed for weeks or months in the initial phase of treatment so any alterations to ATD dose should primarily be based on thyroid hormone concentrations.

CBZ or MMI will reduce thyroid hormone concentrations satisfactorily in most patients and the hope at that stage is that the young person is one of the minority who will enter remission if ATD is stopped. Remission following a course of ATD is currently the only route to a life off medication because the only other regularly-used treatment modalities in children and adolescents are surgery (total thyroidectomy) or radioiodine – both of which are associated with the need for long term thyroid hormone replacement. Unfortunately ATD therapy tends to be associated with more adverse events and tends to be less efficacious in terms of facilitating longer term remission when compared to adults.

### ATD side-effects

**Fig. 1:**
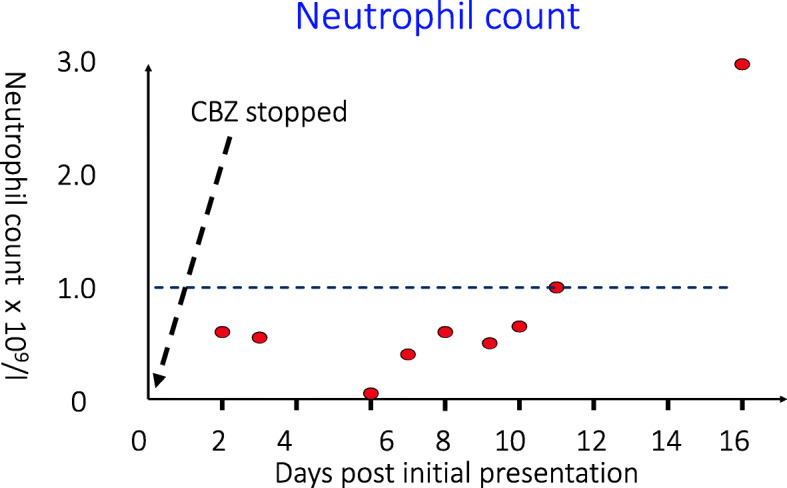
shows the neutrophil count in a patient who had been receiving antithyroid drug (carbimazole, CBZ) for several years. She experienced fever and a severe sore throat and stopped her antithyroid drug 2 days prior to seeking help. Her white cell count was then checked and she subsequently became severely neutropenic. She was treated with intravenous antibiotics prior to neutrophil count recovery. A link with CBZ was thought likely

ATD side-effects are relatively common in children and are observed in 20–35% of patients compared to around 17% of adults [17–20]. The figures reported will reflect the nature of the underlying study with a prospective design generating different figures to retrospective reporting systems. Common side-effects include rash, pruritus and arthralgia – all of which tend to settle with time. Patients and families need be educated about the key life-threatening side effect of CBZ/MMZ which is agranulocytosis. This affects less than 1% of patients but management is complicated by the fact that a low white cell count can be a feature of inter-current infection and can also be found at diagnosis prior to ATD therapy [21]. A neutrophil count less than 0.5 necessitates stopping ATD but values above 0.5 can be monitored closely to see if the white cell count recovers spontaneously. ATD-related agranulocytosis usually occurs in the first months of therapy [22] but can still arise after many years of treatment [23] and the potential for longer-term ambivalence regarding this possibility needs to be borne in mind when patients are reviewed in clinic (*see* Fig. 1). Symptoms and signs suggestive of agranulocytosis include mouth ulcers, pharyngitis and fever. A prompt, time-efficient way of ensuring that families can have a white count checked at short notice is important. Regular testing of white cell counts as part of GD management is a controversial topic with no strong evidence to suggest benefit, in part because of the rapid onset of agranulocytosis. Another rare but important side-effect is Stevens Johnson syndrome which necessitates immediate ATD cessation. PTU can cause hepatic necrosis which is devastating and requires liver transplantation whereas CBZ /MMZ can result in cholestasis that – reassuringly – resolves when the drug is stopped. It is wise to check a full blood count and liver function before commencing ATD so that any subsequent abnormalities are easier to interpret.

An occasion when PTU administration may be required is in the young person with GD is in the hyperthyroid patient who has developed side-effects to CBZ (other than agranulocytosis) and who needs to be rendered euthyroid for surgery (thyroidectomy). However even in these circumstances PTU is frequently not required because a course of iodine will normalise free T3 levels within 7 days in most patients as discussed below.

### Agranulocytosis mechanism

The underlying mechanism behind the development of agranulocytosis is unclear but this may reflect either the oxidative property of the ATD (mediated by myeloperoxidase and cytochrome P450) that then generates reactive metabolites that induce the apoptosis of granulocytes or an immune-mediated process with the development of anti-neutrophil cytoplasmic antibodies (ANCAs) that result in the reduced production, destruction and apoptosis of neutrophils [24]. Although there are no strong clinical or biochemical predictive factors to identify those individuals at risk genome wide association studies in Taiwanese and Caucasian adults suggest that HLA subtypes B*38:02, DRB1*08:03 and B*27:05, are susceptibility loci for ATD-induced agranulocytosis [25]. Carriers of a rare NADPH oxidase 3 (NOX3) variants were demonstrated to have increased apoptosis of methimazole-treated granulocytes [26] and genetic analysis may be a component of the personalised management of ATD in GD in future.

### Weight gain in patients on ATD

An additional complicating factor in the young person is the fact that they may quickly realise that restoration of a euthyroid state can be associated with rapid weight gain which is not a good recipe for concordance. There are a number of publications in the paediatric and adult literature documenting the link between GD treatment and excessive weight gain [27].

### Thyroid storm

Thyroid storm or crisis is a rare consequence of thyroid hormone excess that can be present at the time of diagnosis or be precipitated in someone with GD who is not on ATD by surgery or radioiodine (RAI) therapy. The patient may be tachycardic and hyperthermic with profound agitation and an altered mental state. A thyroid crisis is a medical emergency requiring management in a high- dependency or intensive care setting. Key elements of treatment include the administration of iodine (e.g., potassium iodide solution) as well as glucocorticoid in addition to ATD and a beta-blocker. ATD and iodine block thyroid hormone synthesis and secretion, glucocorticoids inhibit peripheral conversion of the (inactive) prohormone T4 to metabolically active T3 and beta-blockers attenuate the peripheral adrenergic actions of T3.

### Block and replace or dose titration ATD

**Fig. 2:**
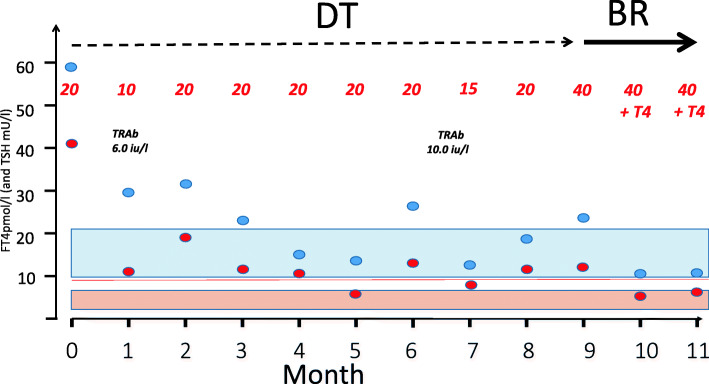
shows the biochemistry of a patient with clinically severe GD managed with dose titration (DT) ATD and then block and replace (BR) ATD. The blue circles represent the free T4 concentrations and the red dots the TSH concentrations. The figures in red refer to the CBZ dose (mg). The instability of the thyroid hormone concentrations in this patient with severe GD ultimately resulted in a change from DT to a regimen comprising a larger dose of CBZ as part of a BR regimen. This clinical case and others like it support the use of a BR strategy but underline the importance of obtaining data from randomised trials (TRAb – thyroid receptor antibody titre; Free T4 normal range 10 to 21 pmol/l; TSH normal range 0.5 to 6 mU/l)

**Fig. 3:**
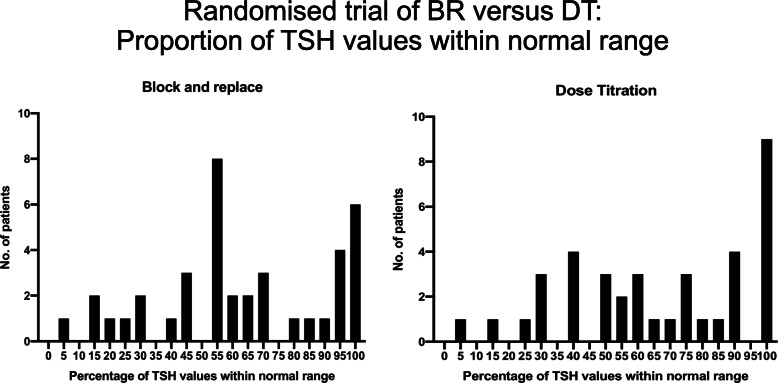
Figure demonstrating the proportion of TSH data within the reference range in young people with thyrotoxicosis randomised to a trial comparting a BR and DT ATD regimen (reference [31]). The treatment interval was between 6 and 36 months post-diagnosis with visits scheduled every 3 months. The figure demonstrates no significant difference between the 2 regimens in terms of the proportion of TSH values within the reference range

The adult literature suggests that the best way to administer ATD is to titrate the dose against prevailing thyroid hormone concentrations (dose titration approach or DT) although some clinicians advocate completely abolishing endogenous thyroid hormone production and starting thyroxine replacement (block and replace or BR). A key underlying premise for those who advocate BR is that it is easier to replace thyroxine in an appropriate quantity in the ‘blocked’ Graves’ patient than it is to adjust the ATD dose in a way that renders the hyperthyroid patient euthyroid. The American Thyroid Association guideline that recommend the DT strategy does so on the basis that the likelihood of side effects is reduced [28, 29] but some clinicians continue to feel that biochemical stability is better with BR [30] and this strategy is still used regularly by paediatricians in the UK [31, 32]. The potential benefits of one approach versus the other may be different in childhood because the underlying disease tends to be more severe and because the young, growing person is changing in size with an associated change in thyroid hormone requirement. There have been a number of useful additions to the literature in recent years since the ATA published their guideline. A report from Italy suggested that BR may indeed be associated with greater stability in the young person but this was not a randomised clinical trial and the switch from DT to a BR regimen occurred when patients had already received ATD for some time and so the disease severity and underlying antibody titres were likely to be less profound by this stage [33]. The biochemical course of a patient managed locally by DT and then BR is shown in Fig. 2 and illustrates the importance of randomised trial data when assessing the merits of one regimen versus the other. A retrospective audit in adult patients did not see any clinically meaningful difference in biochemical control although there was a small but statistically significant difference in the number of follow-up visits favouring BR [34]. A recent randomised trial of DT versus BR in young people has shed further light on this area [35]. The primary focus of this clinical trial was biochemical control as determined by TSH concentrations within the reference range beyond the first 6 months of treatment. The interval between clinic visits was fixed although clinicians could still see patients between visits according to clinical need. The expectation that biochemical control might be better with individuals on BR was not observed. Whilst the study was underpowered the likelihood of a beneficial effect of BR on biochemical control being overlooked because of this was remote and there were more adverse events in the BR group [35]. The distribution of TSH concentrations in patients from this trial are shown in Fig. 3. The fact that there is no meaningful improvement in biochemical control on BR in most patients therefore fits with the adult literature and is important information when discussing management with a family where there is a young person with newly diagnosed GD. With GD of mild to moderate severity, DT therefore remains the preferred option although it is possible there may yet prove to be an advantage in moving to a BR strategy if the disease is severe and biochemical control is challenging in the initial months post-diagnosis. The associated discussions are likely to be quite detailed and are best led by a paediatric endocrinologist who is familiar with the nuances of ATD treatment. Most ATD side effects occur at an early stage and if patients are moved from DT to BR after the first few months then most will be able to tolerate the increase in ATD dose.

### How to manage the patient who does not respond to ATD

If the patient does not become euthyroid despite a substantial dose of ATD then it is important to check that the medication has been taken as scheduled. It is possible to increase the dose of CBZ up to 1.3 mg/kg/day but it is important to make sure that potential aggravating factors such as a substantial iodine intake are not complicating the clinical picture. This is an unlikely scenario in childhood. The occasional patient will still, for poorly defined reasons, fail to become euthyroid and in these circumstances definitive treatment with RAI or blocking with iodine prior to surgery (total thyroidectomy) are potential ways forward.

### Treatment duration

The traditional approach in the young patient with GD has been to treat for approximately 2 years but increasingly longer periods of therapy are being advocated [36]. With this in mind a recent article from Iran suggested that after 10 years of therapy remission rates were as high as 80% although caution should be exercised when discussing this study with families because of the small numbers of patients involved and the potential for underlying differences in patient characteristics at baseline [37]. Factors favouring longer term therapy include the low remission rate after a short, 1- 2 year course of treatment, the fact that the risk of adverse events falls with time and the potential for an increased likelihood of remission. Reports highlighting how well longer term low-dose ATD therapy is tolerated in the young, coupled with reports of remission in 50% or more when ATD is eventually stopped are therefore starting to refine management in patients at the mild to moderate end of the disease spectrum [36–38]. Although the likelihood of disease remission appears to increase with ATD duration to a point, the precise relationship between duration and remission rate is unclear. This is in part because there is a relative dearth of data in GD patients treated for a lengthy period of time. A key consideration when interpreting studies of ATD therapy is whether an ‘intention to treat’ analysis was performed. The proportion of patients in remission when calculated as a percentage of all diagnoses will usually be smaller than the percentage calculated as a proportion of patients with a known outcome.

Before ATD is stopped it is important to check TRAb titre because the likelihood of remission is greater if they are no longer elevated. If TRAb are still measurable then remission is extremely unlikely and ATD therapy should be continued. In our experience most people with GD will have negative thyroid receptor antibodies after 12–18 months of ATD therapy. Further progress may well be made in future if immune markers can be used to complement clinical criteria and provide greater confidence around who will and who will not remit after ATD therapy.

### Stopping ATD

When ATD is stopped it is important to make sure that the patients and family are aware of what to do should thyroid hormone excess recur. Patients should have their thyroid function checked routinely every 3 months after stopping ATD for the first year but will need to have thyroid function checked in the interim if they are concerned about signs or symptoms suggestive of relapse. If the patient is euthyroid at the end of 12 months then the likelihood of relapse is small and thyroid status can be checked less frequently. If the patient is euthyroid 2 years after stopping ATD then it is feasible to discharge the patient back to the general practitioner although bloods will still need to be checked on an annual basis because of the possibility of late relapse but also because of the possibility of autoimmune hypothyroidism [39].

### Alternative strategies (see Table 1)

One of the future areas for research in ATD management is to focus in greater detail on those patients with more severe disease who are less likely to respond to ATD in the longer term. Perhaps these patients may ultimately benefit from the newer immunomodulatory strategies such as the use of biologics, TSHR antibodies and peptide therapy [40–42]. Although iodine is typically used to render patients euthyroid prior to surgery there is an evolving experience of using this as a long-term therapy on the basis that many patients will not ‘escape’ [43].

**Table 1:** GD management – the future

• The relationship between ATD duration beyond 5 years and likelihood of relapse needs to be established in greater detail.	
• The baseline immune and genetic factors that may impact on the likelihood of remission need to be explored in more detail. This may help to predict outcome.	
• Patients can potentially be stratified as at low or high risk of an adverse reaction to ATD including agranulocytosis with associated alterations in surveillance program.	
• The potential role of long-term iodine therapy needs to be explored in more detail.	
• The role of existing and novel immunomodulatory agents needs to be explored.	

### When to opt for definitive treatment (see Table 2)

There are a number of reasons why the young person may opt for definitive treatment. The first and most obvious reason is because of ATD side-effects. Typically this would be either neutropenia although troublesome rashes and Stevens-Johnson would also be criteria for stopping ATD therapy and opting for thyroid gland removal (surgery) or destruction (RAI). Some clinicians are wary about treating young people with ATD who, perhaps because of learning disability, are unable to report symptoms such as a sore throat or fever. These patients may benefit from early definitive treatment on the basis that thyroid thyroxine replacement is relatively straight-forwards and safe. Some young people will also view the prospect of a life on thyroid hormone replacement as opposed to ATD favourably because the regimen is generally simpler with no need to visit hospital or the general practice for a full blood count check when unwell. A move to long-term thyroid hormone replacement as opposed to ATD may also appear attractive for those patients who are due to leave home or travel abroad for a lengthy period of time. If a patient can no longer take CBZ because of side effects other than neutropenia then a short course of PTU can potentially be used to render the patient euthyroid prior to thyroidectomy although iodine would be the preferred option. The disadvantage of iodine is that there is a potential for the patient to become hyperthyroid at a later date and so the patient wanting thyroidectomy will usually need to have surgery within a certain time frame before ‘escape’ from biochemical control can occur. The advantages and disadvantages of surgery (total thyroidectomy) and RAI are summarised in Table 2.

**Table 2:** Advantages and disadvantages of surgery (total thyroidectomy) and RAI

**Advantages and Disadvantages of Radio-iodine**	
Advantages include:	
• Simple	
• No scar	
• No surgical risk	
Disadvantages include:	
• Best avoided in the young, pre-pubertal child if possible	
• Best avoided in large goitres where second dose may be needed	
• Need to follow safety guidance post Rx with implications in terms of school, contact with other family members etc	
• Risk of crisis (very small)	
• Can excacerbate eye disease – glucocorticoid cover may be needed	
• Fluctuating thyroid status post administration – can be addressed by a period of BR therapy with ATD stopped after ~ 4 to 6 months	
• Potential need for second dose – reduced likelihood if larger dose or (potentially) if dosimetry used	
• Must be avoided if there is a risk of pregnancy	
• Potential longer term neoplasia risk	
**Advantages and Disadvantages of surgery (total thyroidectomy)**	
Advantages include:	
• Immediate resolution of hyperthyroidism	
• Removes goitre	
Disadvantages include:	
• Anaesthetic risks	
• Well recognised surgical risks eg bleeding	
• Scar	
• Long term hypoparathyroidism	
• Voice change (damage to recurrent laryngeal nerve or superior laryngeal nerve’s external branch)	

In the case of female patients the potential link between history of GD and neonatal thyrotoxicosis needs to be discussed.

### Personalized ATD treatment

Ultimately the choice of treatment rests with the young person and their family but there are few absolutes. A range of factors may be associated with an increased likelihood of remission such as older age (adolescence versus childhood) and a less profound clinical, biochemical and immunological disturbance at presentation [44] but there are still young patients with a high likelihood of relapse who remit after a relatively short course of ATD and patients with a high likelihood of remission at diagnosis who may relapse after many years of therapy. The young person and their family will need to be aware of this uncertainty.

### Longer term outcome

The presence of TPO antibodies in autoimmune thyroid disease is associated with a long-term risk of hypothyroidism and their presence in GD is a key reason why patients are at risk of hypothyroidism after ATD is stopped [39]. This issue has not necessarily received the attention it deserves because Graves’ disease remission does not necessarily mean that the patient will be medication-free in the long-term. The patient who is TPO antibody positive 1–2 years post antithyroid drug still needs monitoring to make sure that they remain euthyroid. There is relatively little data on quality of life in people with GD although a recent study has shown that young Graves’ patients have a lower total QoL score compared with a healthy cohort [45]. Individuals who chose definitive treatment with RAI or surgery did not regret this decision.

## Conclusions (see Table 3)

GD is a frustrating condition for many young people and their families. The diagnosis is not always made quickly after symptoms and signs develop and the disorder can have a major impact on educational progress. Treatment can be tailored to the individual according to likelihood of remission although long term ATD is a realistic option even in the context of a high likelihood of relapse on the basis of presenting features. The DT strategy remains the preferred approach when administering ATD and the possibility of remission / stopping ATD is realistic if patients are on a very low dose of ATD with a normal TRAb titre. The potential role of novel immunomodulatory agents is being explored.

**Table 3:** Summary of recent developments in ATD administration and long term outcome

• PTU should be avoided in the young patient with thyroid hormone excess.	
• Long term ATD therapy with CBZ or MMI in excess of 3 years duration is a realistic option for young patients with GD who are at high risk of relapse.	
• Data from a randomised trial indicates that DT ATD therapy is associated with similar biochemical control when compared to BR.	
• Weight gain can be a major problem in patients with GD starting ATD treatment. This should be discussed with the young person and their family at an early stage.	
• Patients in remission following ATD need monitoring because of the potential for autoimmune hypothyroidism as well as relapse.	

## Data Availability

None.

## Abbreviations

GD: Graves’ disease
CBZ: Carbimazole
MMI: Methimazole
PTU: Propylthiouracil
ATD: Anti-thyroid drug
BR: Block and replace
DT: Dose titration
TRAb: Thyroid stimulating hormone receptor antibodies
TSH: Thyroid stimulating hormone
TSHR: Thyroid stimulating hormone receptor
TPO: Thyroid peroxidase antibodies
CTLA4: Cytotoxic T-lymphocyte-associated protein 4
ANCA’s: Anti-neutrophil cytoplasmic antibodies
NOX3: NADPH oxidase 3
HLA: Human leukocyte antigen
